# Complications of Self-Injected Facial Fillers: A Treatment Conundrum in the UK

**DOI:** 10.1155/2019/2041839

**Published:** 2019-05-22

**Authors:** Jessica Blanchard, Julia Palmer, Enamul Ali, Leo Cheng

**Affiliations:** Department of Oral and Maxillofacial Surgery, Homerton University Hospital, Homerton Row, London E9 6SR, UK

## Abstract

There has been a rise in nonsurgical cosmetic procedures seen within the UK population in the past decade. A change in legislation has placed restrictions on the distribution and provision of such treatments. Therefore, patients may seek alternative methods to bring about a change to their appearance, such as self-injection of a filler. Complications may include oral ulceration, foreign body tissue reaction, and infection due to a lack in sterility during injection. Such presentations may mimic that of oral cancer and can lead to misdiagnoses and undue cost to the National Health Service. This case highlights the common features leading to correct identification of patients self-injecting with facial fillers and discusses the controversy surrounding the economic aspects of their care. We would like to report one such case presenting to our oral and maxillofacial surgical unit.

## 1. Introduction

Social media pressures and an increased trend of the aesthetically conscious have resulted in a rise of patients seeking dermal fillers to alter their facial appearance. Over the past 18 months at Homerton University Hospital, there has been a notable increase of patients presenting with adverse reactions to dermal fillers. This report outlines one such case.

Due to the changes in UK legislation regarding the provision of nonsurgical cosmetic procedures, an increased number of patients are self-injecting dermal fillers bought over the internet. Often these patients are seen in A&E or by their general medical practitioner following complications, as there is no defined pathway for their care.

## 2. Case Presentation

A 24-year-old female patient presented to the Department of Oral and Maxillofacial Surgery at Homerton University Hospital in East London, UK, with a 4-week history of suspicious upper lip swelling. This patient was referred by her general practitioner on the urgent head and neck cancer referral pathway.

The patient's primary concern was the sudden onset of a large upper lip swelling with associated pain. The patient was not forthcoming and defensive when questioned regarding precipitating factors or possible trauma to the region. She was medically fit and healthy with no risk factors for oral cancer, such as smoking and alcohol consumption [1].

**Figure 1:**
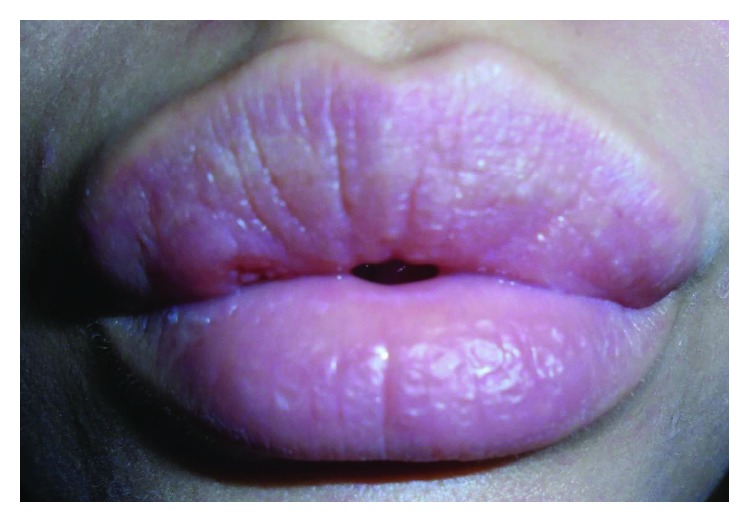
Anterior view of patient on presentation.

**Figure 2:**
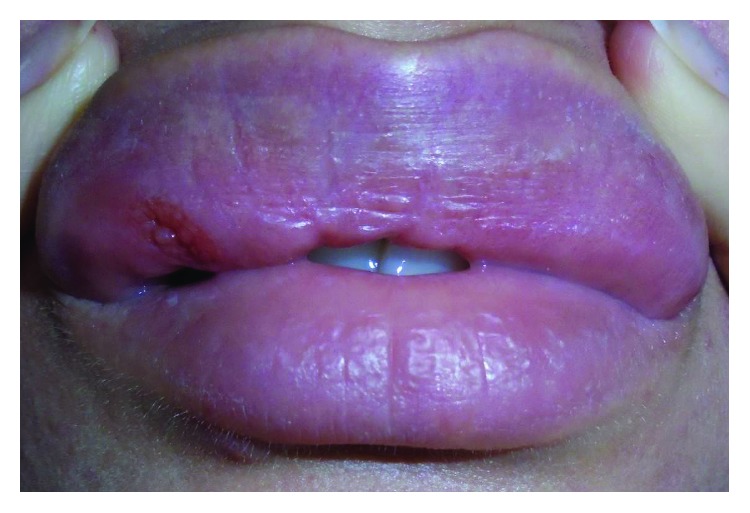
Anterior view of patient showing ulcerated region and puncture wound.

On extraoral examination, the patient appeared to be well with no clinical signs of systemic illness. In general, the upper lip was disproportionately enlarged with an incompetent lip seal (Figure 1). On close examination, a 2 cm × 1 cm firm, erythematous, swelling was noted in the upper right lip. The lesion was not encroaching on the midline and there was no associated cranial nerve deficit or lymphadenopathy. At the vermillion border, there was a puncture wound on a background of traumatised tissue adjacent to the firm swelling (Figure 2). The lesion was painful on palpation with no suppuration, induration, or ulceration noted. Intraoral examination revealed a concurrent firm swelling within the labial sulcus. Dental health was unremarkable, and there were no signs of systemic infection.

Upon revision of the patient's electronic health-care record, it was noted that the patient had attended the Accident and Emergency Department at the Homerton University Hospital four weeks prior. The clinical clerking from this visit showed the main complaint to be swelling of the upper lip following self-injection of a dermal filler, purchased over the internet. When questioned again directly by the OMFS team regarding the true mode of injury, the patient revealed that she had purchased the filler material over the internet. The patient was uncertain of the name of the website or the filler material. She was unaware if the material was nonpermanent (temporary), semipermanent, or permanent [2].

## 3. Differential Diagnoses

1. Dermal infection
2. Early granulomatous reaction secondary to filler injection
3. Traumatic lesion
4. Type IV allergic reaction

## 4. Treatment

The patient was reassured and removed from the urgent two-week wait pathway as malignancy was excluded.

Due to the history and clinical appearance, the provisional diagnosis of granulomatous reaction secondary to dermal filler injection was made. The patient was given the following treatment options: a conservative approach of monitoring via clinical photography and, secondly, administration of intralesional hyaluronidase. Hyaluronidase is an enzyme which cleaves the peptide bonds within the hyaluronic acid [3]. However, due to the unknown nature of the hyaluronic acid injected, it was deemed that injection of hyaluronidase may be unpredictable, and thus the patient was advised against this option. A further option of surgical excision was outlined to the patient. The risks of surgical intervention including infection, altered sensation, and scarring were discussed. The patient opted for conservative management initially.

## 5. Outcome and Follow-Up

**Figure 3:**
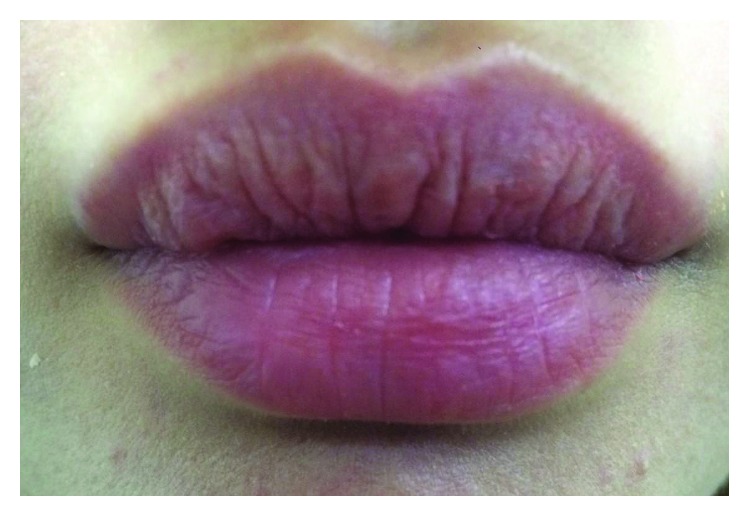
Anterior view of patient at follow-up, one week post-management.

The patient was reviewed one week after initial presentation. Clinically, there was a generalised reduction of oedema and pain. The patient returned for a review appointment at two months, and at this appointment the swelling had completely resolved; the patient was thus discharged.  Figure 3 depicts the patient at one week postmanagement. It can be seen from this postmanagement photograph that there is a reduction in swelling with the resolution of the punctum.

Upon reflection, it may have been beneficial to undertake a more thorough work-up to aid diagnosis. An incisional biopsy of the lesion at initial presentation, FNA, or even a plain film radiograph could have been taken. However, following an initial discussion with the patient regarding treatment options, it was clear that surgical intervention and its associated risks was not favoured by the patient. At the one-week review appointment, had there not been a resolution of symptoms, biopsy and further surgical management would have been considered.

£99 was the cost of the initial A&E encounter, £153.06 for the first clinic appointment with OMFS, and £71.11 for the follow-up clinic appointment. Therefore, the total cost to the National Health Service for these three clinical encounters was £323.17. The authors note that there have been cases where further investigations (US+FNAC, MRI, and incisional biopsy) have been undertaken due to the lack of transparency on the patient's behalf, thus further increasing costs to the health service. In this case, the diagnosis and management were aided by the ready access to the patient's electronic record from 4 weeks prior. The authors are aware that other hospitals do not often have this accessing ability; therefore, a precise history is vital.

## 6. Discussion

Market analysis has shown that the cosmetic surgery industry is growing rapidly. It is thought that nonsurgical procedures account for more than 90% of the market with most being undertaken on younger female adults aged 16-24 [4]. These procedures are predominantly performed outside of the United Kingdom's “National Health Service,” within the private sector. It is thought that the increased demand for cosmetic procedures has been driven, in part, by increased social pressures placed on the younger generation via social media. Not only has social media and the internet caused cosmetic procedures to become normalised, it has also provided a platform for the treatment to be marketed [5].

Further to this, new legislation introduced by HEE in 2016, has outlined a change in regulation to those providing nonsurgical cosmetic procedures [6, 7]. This has ensured that dermal fillers can no longer be injected by those not registered with a health-care body. Significantly, a rise in the presentation of patients who have self-administered dermal fillers has been seen within our own clinical practice and others [8].

The advances of technology have opened the doors to an unregulated and easily accessible forum where members of the public can buy self-injectable fillers. Alarmingly, the general public awareness to the risks of dermal fillers appears to be lacking. Unfortunately, the authors were unable to identify the exact filler used by this patient as this information was unknown to the patient herself. However, we were able to identify and contact an online vendor of a semipermanent injectable dermal filler. We posed as an interested buyer, and the vendor was eager to sell the filler with no restriction or advance warning of the risks involved. Just as it is easy to access such treatments, perhaps legislation needs amending to carry warning signs. In addition, it is proposed that providers give mandatory complication leaflets so that the general public are more aware of the risks.

Acute complications of dermal filler injection, as experienced by this patient, can include oedema, erythema, ulceration, suppuration, and infection. However, more severe acute adverse events can occur, such as vascular compromise resulting in necrosis and blindness [9, 10]. Chronic complications can include long-standing granulomatous reaction, facial deformity, and requirement for surgical removal. The patient considered in this report experienced the acute complications of injectable dermal fillers and was reviewed at three months to ensure that no chronic complications had occurred. This article has primarily concentrated on the complications of dermal fillers. There are, however, numerous other facial rejuvenation treatments available, each with their own specific complications [11, 12].

There is no established pathway within the United Kingdom for the treatment of facial nonsurgical cosmetic complications which can be caused by both self-administration or health-care-professional administration [13]. The National Health Service is yet to publish data regarding the exact number of cosmetic procedure complication cases presenting to the National Health Service annually, and therefore, no estimate of this total cost can be made. However, an independent review undertaken by the UK government's “Department of Health” in 2013 states that the cosmetic intervention sector is “growing rapidly” with an increased incidence of complications associated with both surgical and nonsurgical procedures seen. This document highlights serious concerns about the cosmetic procedure industry and proposes much tighter and rigorous regulation for nonsurgical cosmetic procedures. However, the review did not outline a proposed pathway for the management of complications caused by surgical or nonsurgical cosmetic procedures [14]. These patients may present to the National Health Service through A&E or their general medical practitioner. Health-care professionals should be more aware of these patients. Identification of these patients is paramount; patients may not be forthcoming with regards to the history and the mode of injury. Social risk factors for oral cancer should also be taken into account.

Worldwide, there has been a reported increase in the prevalence of patients experiencing complications, secondary to the injection of dermal fillers [15]. This correlates with an increase in the undertaking of dermal filler injections. The American Society of Plastic Surgeons reported a 3% rise in patients undergoing dermal filler enhancement [16].

Currently, clinicians within the UK do not receive formal training regarding cosmetic procedures available and their potential complications. The rise in popularity of nonsurgical cosmetic procedures such as dermal fillers has enviably brought with it an increased prevalence of complications, and the medical curriculum should be altered to educate clinicians on the recognition and management of these patients. This poses a challenge as cosmetic surgery itself is not a discrete area of surgical practice but a component of a number of surgical specialties. Therefore, there is no individual professional organisation that is responsible for setting standards which could be used in training and an adjustment to training would be required over multiple specialties.

Further to this, is it within the remit of the NHS to provide treatment for those patients with complications from private cosmetic procedures? As health-care professionals, it is our duty to provide acute management of complications [17]. The case described within this report is not a stand-alone case. As the number of people within the UK seeking nonsurgical cosmetic procedures has increased, so too has the number of patients presenting to the National Health Service for management of complications of these procedures. As there is no defined pathway for the treatment of these patients, a conversation needs to be had between the general public, clinicians, and the government regarding what is expected for the long-term care of patients suffering from the complications of nonsurgical cosmetic procedures. If it is to be managed within the National Health Service, alteration to the training of clinicians needs to be made and the source of funding required to sustain the increased cost to the health service must be clarified.

## 7. Learning Points/Key Messages

1. Patients with facial dermal filler complications may be initially misdiagnosed as oral cancer. Therefore, their identification is paramount
2. Changes to the UK regulation in 2016 has led to an increased prevalence of facial dermal fillers self-injected by patients
3. Clinical features may include oedema, erythema, ulceration, suppuration, and pyrexia, with possibility of a localised puncture wound. These patients are commonly young and may not have the social risk factors for oral cancer
4. There is currently no established pathway for the management of complications related to facial dermal fillers. Financial implications to the NHS need to be taken into account
